# Mega Virchow–Robin

**DOI:** 10.5334/jbsr.3733

**Published:** 2024-09-17

**Authors:** Leonor De Almeida Moreira Cameira de Abreu, Sophia Chkili, Denis Tack

**Affiliations:** 1 Department of Radiology, Epidura La Madeleine, Rue Maria Thomée, 1, 7800 Ath, Belgium; 2 Department of Radiology, Epidura La Madeleine, Rue Maria Thomée, 1, 7800 Ath, Belgium; 3 Department of Radiology, Epidura La Madeleine, Rue Maria Thomée, 1, 7800 Ath, Belgium

**Keywords:** perivascular spaces, magnetic resonance imaging

## Abstract

*Teaching point:* Perivascular spaces, also known as Virchow–Robin spaces, are fluid-filled spaces that surround the vessel walls from the subarachnoid space through the brain parenchyma.

## Case Presentation

A 61-year-old man with a history of hypercholesterolemia, arterial hypertension, and new-onset type 2 diabetes was referred to the Radiology department for major ataxia and severe cognitive trouble.

Although cooperative and well oriented, neurocognitive evaluation revealed a globally impaired cognitive profile, with severe deficits in memory, executive, and attention functions.

A magnetic resonance imaging (MRI) was performed to further investigate the cognitive decline.

**Figure 1:**
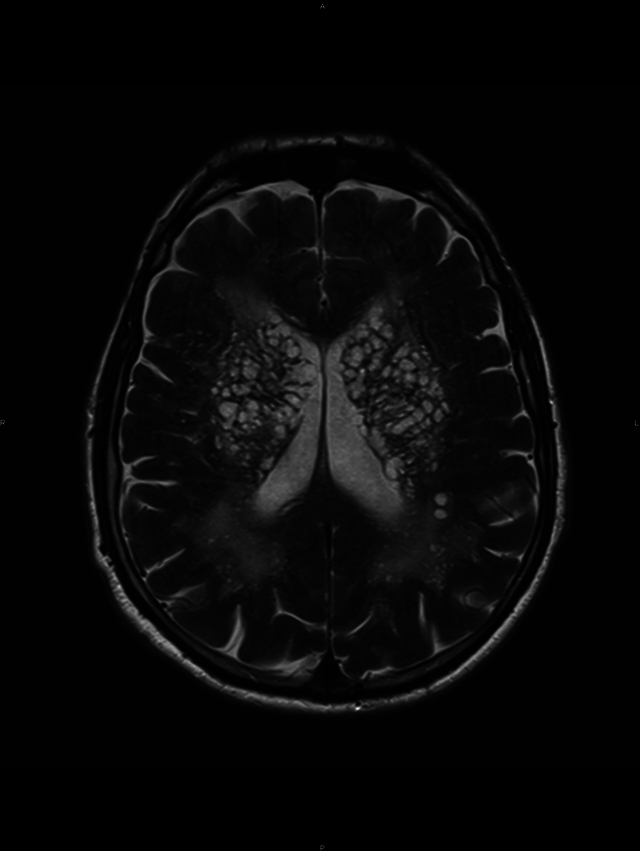
High signal in T2 weighted sequence.

**Figure 2:**
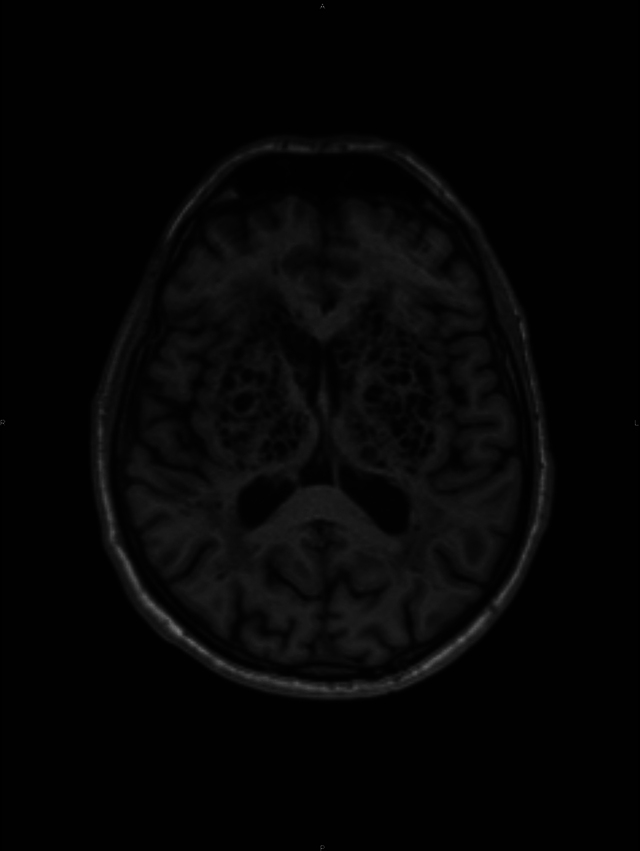
Low signal in T1.

**Figure 3:**
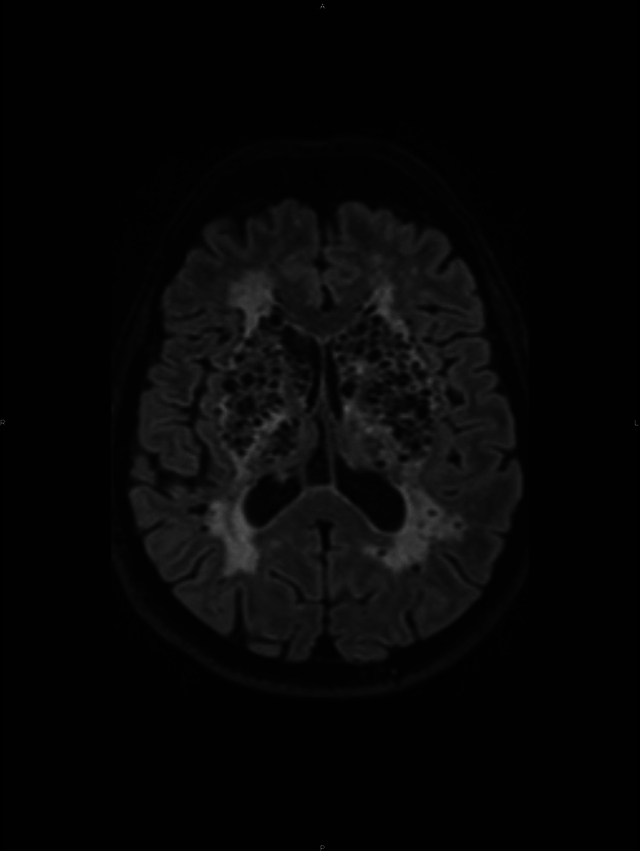
T2 fluid-attenuated inversion recovery.

A conglomerate of multiple, symmetric, well-circumscribed round/oval lesions. These lesions presented high signal in T2-weighted sequence (Figure 1) and low signal in T1 (Figure 2) and in T2 fluid-attenuated inversion recovery (Figure 3). No diffusion abnormalities were identified. No enhancement was detected after contrast media administration.

No mass effect was originated by the presence of these brain formations.

Additionally, marked supra-tentorial microvascular periventricular leukoaraiosis and global cortical atrophy were seen. The lesions followed cerebrospinal fluid signal characteristics.

Therefore, these findings are consistent with dilated perivascular spaces, also called dilated Virchow–Robin spaces.

## Discussion

A German pathologist, Rudolf Virchow, and French anatomist, Charles Philippe Robin, in the XIX century gave their names to the Virchow–Robin spaces [1].

Perivascular spaces are microscopic, usually not visible on MRI. When visible they are considered dilated. They surround the vessel walls in their course from the subarachnoid space through the brain parenchyma. Although they can appear in any location of the brain, they tend to be located around the lenticulostriate arteries.

The dilation of Virchow–Robin spaces was described by Durant-Fardel, in the middle of the XIX century [1]. These dilatations are regular, round, or oval cavities, with a well-defined and smooth margin following the path of penetrating arteries and have similar cerebrospinal fluid signal characteristics on all MRI sequences.

Dilated Virchow–Robin spaces typically present in three characteristic locations: Type I Virchow–Robin spaces appear along the lenticulostriate arteries penetrating the basal ganglia through the anterior perforated substance. Type II Virchow–Robin spaces are found along the paths of the perforating medullary arteries as they enter the cortical gray matter over the high convexities and extend into the white matter. Type III Virchow–Robin spaces usually appear in the midbrain [1].

Although discovered more than a century and half ago, mechanisms underlying expanding Virchow–Robin spaces are still unknown. Dilated perivascular spaces are very frequent; nonetheless, they rarely present in such an oversized aspect and abundance.

Knowledge of the signal intensity characteristics and locations of Virchow–Robin spaces helps differentiate them from various pathologic conditions, for instance, lacunar infarctions and multiple sclerosis.
